# Commercialization Report: Modulim Inc.

**DOI:** 10.1117/1.BIOS.3.1.010502

**Published:** 2026-03-28

**Authors:** Amaan Mazhar, David Cuccia

**Affiliations:** Modulim, Inc., Costa Mesa, California, United States

## Abstract

This community report discusses the experience of navigating the complex path of commercializing biomedical technologies, offering lessons for the scientific community and industry stakeholders alike.

## Company Overview and Founding Team

Amaan Mazhar is CEO of Modulim and David Cuccia is Founder/President/CTO. Modulim was founded by a graduate student (Cuccia), two postdocs, and two faculty (Bruce Tromberg and Anthony Durkin) at the Beckman Laser Institute (BLI). Cuccia was the first full-time employee of the company and Mazhar was third, and both have been involved with development (lab + commercialization) of spatial frequency domain imaging (SFDI) technology for over 15 years. Both Mazhar and Cuccia were graduate students and researchers at the founding SFDI lab at BLI.

The company originated over 15 years ago under the name Modulated Imaging Inc., before later rebranding to Modulim to reflect its evolving medical focus. The founding team operated out of a small initial workspace provided by the BLI at the University of California – Irvine (UCI) prior to raising capital and establishing its own space. As the company evolved, Cuccia’s role centered on scientific and technical leadership, while Mazhar’s responsibilities expanded into strategy, operations, and external partnerships, leading to their current CTO and CEO roles.

## Technology Description

Modulim has commercialized both research and FDA-cleared clinical SFDI systems. SFDI is a widefield diffuse optical method that can help the user to assess tissue structure and function. The technology calculates quantitative maps of tissue optical properties (absorption and scattering) that can be used to characterize microcirculatory status (oxygenation and perfusion). These maps provide clinical insights about tissue health that other current assessment tools do not provide. Over time, the system has been refined to improve clinical usability, speed, and workflow compatibility, building on foundational academic prototypes developed during Cuccia’s graduate research.

## Clinical Need and Market

It is estimated that 50% of diabetes patients that get a leg amputation due to a chronic wound have never had a specialist vascular assessment. Modulim’s imaging and software solutions are designed to help healthcare teams assess tissue health at the point of care with the goal of enabling healthcare teams practice proactive management and prevention of these serious complications. Modulim’s products help healthcare teams identify potential microvascular and macrovascular circulatory compromise in patients with diabetes, kidney disease, or peripheral arterial disease. These circulatory complications are a precursor to chronic wounds that lead to limb amputations. The company’s clinical focus has sharpened over time toward scenarios where early, quantitative identification of circulatory compromise can meaningfully change outcomes by introducing effective care earlier. Earlier detection aligns the interests of providers and payers by helping prevent advanced complications such as amputations, which carry both high morbidity and high cost.

Modulim’s SFDI technology and tissue assessment is platform by nature and the team is working on future markets through collaborative partnerships. An example of this is using SFDI to assess burn severity at the point of care to help determine triage need—this is supported by an ongoing partnership with the military. Other work using embodiments of the Modulim’s research devices have included collaborations in surgery (i.e., skin flaps), dermatology, applications (skin cancer, skin health), and oncology (tumor margins).

## Commercialization Stage and Sales

**Fig. 1:**
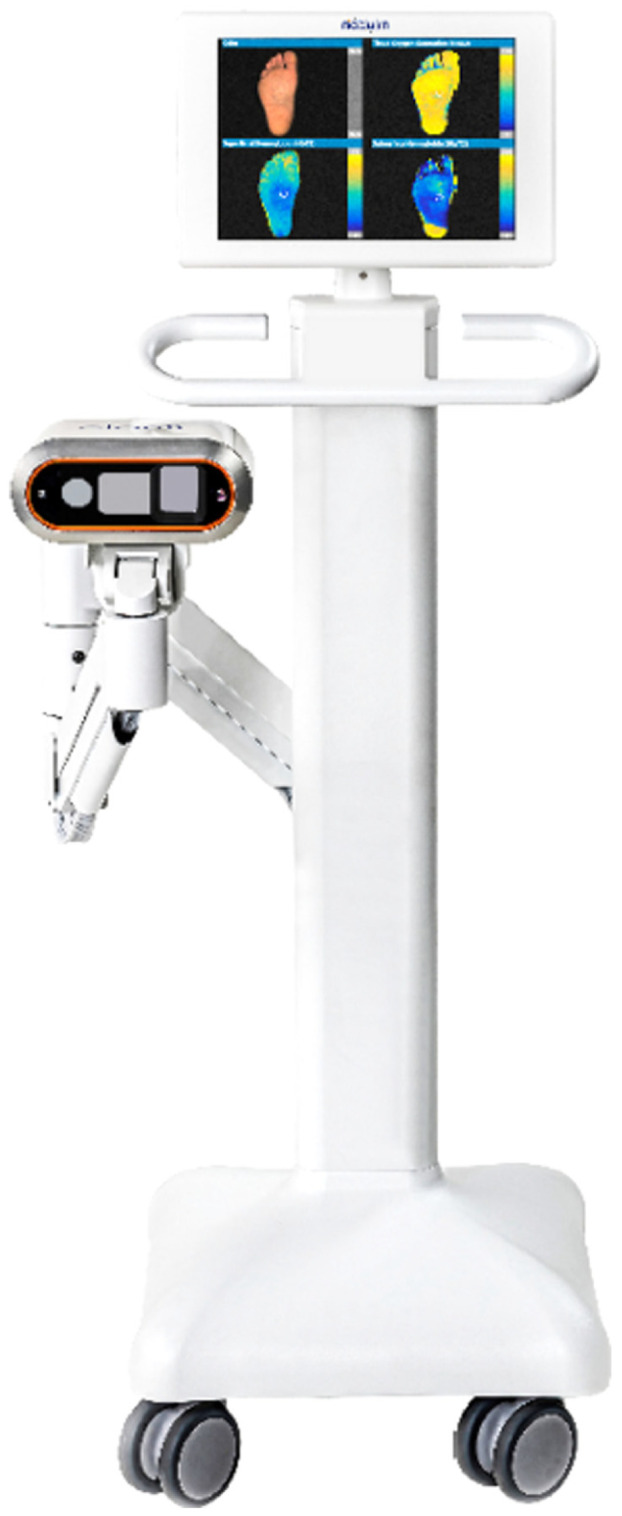
The Clarifi Imaging System is an FDA-cleared device to determine oxygenation levels in superficial tissue for patients with potential circulatory compromise. Image courtesy of Modulim.

Modulim has both research and clinical products. The research product is primarily sold to academic researchers and collaborators with the benefit of standardizing SFDI data collection, calibration, and processing so SFDI can be used for various research applications. The clinical product, the Clarifi Imaging System (Fig. 1), is FDA-cleared and sold to healthcare groups that are assessing potential circulatory compromise in their diabetes and chronic kidney disease, and peripheral arterial disease patients.

Feedback from early adopters played a critical role in refining the clinical system, as direct user experience highlighted necessary improvements in workflow, usability, and data review tools. This iterative development process helped bridge the gap from research instrumentation to a reliable clinical product.

## Competitive Landscape

The current standard of care for proactive vascular assessment in the limb is a visual check, which is highly subjective, inconsistent, and looking for a late-stage issue (i.e., wound). Other non-imaging technologies like ankle-brachial index (ABI) are used sparingly to identify a subset of potential macrovascular circulatory issues but have degraded performance in the target high-risk diabetes patients. Ultrasound is also limited because of the need for specialist skills.

Modulim’s value proposition is to offer an objective technology that works well at the point-of-care and for high-risk diabetes patients. The maps also provide insight over a larger area where wounds often form and help healthcare teams determine if issues are due to underlying macrovascular and microvascular changes.

## University Support and IP Licensing

The core technology was invented at the Beckman Laser Institute (BLI) at the University of California, Irvine (UCI). The technology transfer office at UCI provided support/infrastructure for licensing the technology and the BLI provided space and a collaborative environment during the early days. UCI has created an excellent ecosystem that supports local entrepreneurs/startups and a community that includes access to seed funding. This enabled the transition of SFDI from a prototype system to commercial hardware.

## Funding History

Initial funding for Modulim came from a combination of hardware/software contracts and Small Business Innovative Research (SBIR) grants. In addition to SBIR funding, Modulim generated revenue from research sales/collaborations during the early phases of the company. Once the company transitioned to releasing its first medical product, it was able to raise its first equity financing. Investors have included angel, traditional, and strategic venture capital.

Modulim was able to achieve the first key technical milestones with Phase I/II SBIR funding. These milestones de-risked future regulatory and product milestones which increased the likelihood of equity investments. The SBIR program has been critical to the success of the company.

## Regulatory Pathway

Modulim obtained FDA clearance for its primary device through the 510(k) pathway. This pathway is used for technologies that can show substantial equivalence to existing cleared technologies. Tissue oximeters (probe-based and imaging) are a well-established product for the FDA. In order to obtain 510K clearance, the team established a quality system and executed a regulatory strategy with the appropriate indications for use and intended use to establish substantial equivalence. This was achieved by bringing on the appropriate consultants and team members.

## Lessons Learned

There have been a number of lessons learned in this ongoing journey. Here are three to share:

The first is to get early versions of your product into the hands of users who are willing to provide feedback and validate that there is a market. Even if the product isn’t ready, early adopters offer irreplaceable insight into whether the market exists and there is no substitute for seeing how real users interact with your product so you can iterate.

The second is to be selective with both your team and partners. The right early clinical partners tend to be engaged and collaborative—they can drive adoption and provide references. The right team members are not only good at their job but engaged with building the culture of the organization. It’s important to move on to a new a partner or team member if you aren’t getting what you need.

The third lesson learned is to be consistent in maintaining flexibility as you learn. Market conditions and priorities can change outside of the company’s control. Product-market fit in an evolving ecosystem requires consistent and disciplined commitment/approach to structured learning with a willingness to adapt strategy in response to real-world data.

## Supplemental Information

The interview on which this report is based is available in podcast form at *Biophotonics Discovery: The Podcast*, available wherever you get your podcasts.

